# Arginine Cofactors on the Polymerase Ribozyme

**DOI:** 10.1371/journal.pone.0025030

**Published:** 2011-09-20

**Authors:** Chengguo Yao, Janina E. Moretti, Peter E. Struss, Junaid A. Spall, Ulrich F. Müller

**Affiliations:** Department of Chemistry and Biochemistry, University of California, San Diego, La Jolla, California, United States of America; Max-Planck-Institute for Terrestrial Microbiology, Germany

## Abstract

The RNA world hypothesis states that the early evolution of life went through a stage in which RNA served both as genome and as catalyst. The central catalyst in an RNA world organism would have been a ribozyme that catalyzed RNA polymerization to facilitate self-replication. An RNA polymerase ribozyme was developed previously in the lab but it is not efficient enough for self-replication. The factor that limits its polymerization efficiency is its weak sequence-independent binding of the primer/template substrate. Here we tested whether RNA polymerization could be improved by a cationic arginine cofactor, to improve the interaction with the substrate. In an RNA world, amino acid-nucleic acid conjugates could have facilitated the emergence of the translation apparatus and the transition to an RNP world. We chose the amino acid arginine for our study because this is the amino acid most adept to interact with RNA. An arginine cofactor was positioned at ten different sites on the ribozyme, using conjugates of arginine with short DNA or RNA oligonucleotides. However, polymerization efficiency was not increased in any of the ten positions. In five of the ten positions the arginine reduced or modulated polymerization efficiency, which gives insight into the substrate-binding site on the ribozyme. These results suggest that the existing polymerase ribozyme is not well suited to using an arginine cofactor.

## Introduction

According to the RNA world hypothesis, an early stage of life used RNA both as genome and as catalyst [1], [2], [3], [4] for recent reviews see [5], [6]. The central activity in an RNA world organism would have been RNA polymerization to facilitate self-replication. To recapitulate an RNA world in the lab, RNA polymerase ribozymes were developed and improved in several laboratories [7], [8], [9], [10].

These polymerase ribozymes have a length in the range of 200 nucleotides. Therefore, self-replication would require the polymerization of about 200 ribozyme-encoding nucleotides. However, the best existing polymerase ribozymes favor variants of a single, short template sequence with the length of less than 20 nucleotides. By concatenating multiple copies of this sequence it was possible to extend a primer by 95 nucleotides [10]. However, such a template could not encode a ribozyme. On unrelated template sequences, polymerization reaches usually less than 10 nucleotides, and recent improvements made it possible to polymerize 20–30 nucleotides [9], [10], [11], [12]. However, this is still far below the level required for self-replication. The limiting factor for polymerization efficiency is the ribozyme's weak sequence-independent binding of the primer/template substrate, with a K_M_ in the millimolar range [13]. Some of the sequence independent contacts are hydrogen bonds to template 2′-hydroxyl groups [14]. However, it may be possible to establish additional sequence independent contacts mediated by ionic interactions with the negatively charged phosphodiester groups of the primer/template substrate. To do this, the ribozyme would have to employ a positively charged cofactor.

This positive charge can be supplied by metal ions or by cationic organic molecules. The polymerase ribozyme was originally selected in the presence of 60 mM magnesium ions [7] and different versions were optimized in the presence of 36 to 184 mM free magnesium ions [9], [10]. Because magnesium ions are good ligands for the phosphodiester oxygen anions of RNA [15] the continuous presence of magnesium ions during the evolutionary history of polymerase ribozymes should have found the most beneficial involvements of magnesium ions that increase polymerization efficiency. However, even at the optimal magnesium concentration of 200 mM the binding of substrate is in the millimolar range, suggesting that cations different from metal cations could play a role to improve substrate binding.

In contrast to metal ions the polymerase ribozyme did not encounter cationic organic molecules during its history. Therefore, a potential benefit from those molecules would have gone undiscovered. Specifically, the amino acid arginine carries several advantages over other cationic cofactors. Most importantly, the guanidinium group does not establish a hydration shell in aqueous solution. This helps the binding of negatively charged RNAs because it avoids the enthalpic cost of displacing a hydration shell [16]. Additionally, the guanidinium group of arginine has a pK_A_ of 12.5 [17], maintaining a positive charge at any pH value encountered by the ribozyme. Evidence that these factors benefit RNA binding comes from RNA binding proteins, which use arginine more than any other amino acid at the interface with RNA [18].

How could arginine cofactors compete with the high concentration of magnesium ions that are required by the polymerase ribozyme? In addition to the absence of a hydration shell our experiments carry two designs to help arginine compete with the magnesium ions. First, we decreased the free magnesium ion concentration from 184 mM to 64 mM, which facilitates near-optimal activity, and further down to 24 mM, which allows weak but quantifiable polymerization to occur [19]. Second, we connected the arginine cofactor to the ribozyme via arginine-nucleic acid conjugates, which base pair to the ribozyme and thereby generate a high local concentration of arginine proximal to the binding site. We estimate that the local concentration of the arginine guanidinium group would be at least 50 mM, based on the volume accessible constrained by the length of the linker to the nucleic acid.

In an RNA world, amino acid - nucleid acid conjugates or peptide - nucleic acid conjugates could have served in the roles of cofactors and could have established the first steps in a translation system [20], [21]; see also [22]. The synthesis of such conjugates would have been possible in an RNA world because ribozymes can generate several different types of RNA-amino acid conjugates [23], [24], [25], [26]. One benefit of amino acid - nucleic acid conjugates for an RNA world would have been that less sequence of the ribozyme needs to evolve for pairing a conjugate compared to establishing a binding pocket for the cofactor. This means that the ‘combinatorial cost’ of acquiring a cofactor is strongly reduced, and thereby the evolutionary likelihood of reaching that state is higher.

In this study, arginine was used as a positively charged cofactor for the polymerase ribozyme. An arginine - nucleic acid conjugate was positioned at ten different positions on the ribozyme located near the substrate-binding site. We tested whether the positively charged arginine could be used by the ribozyme to increase polymerization efficiency. However, the arginine did not improve polymerization in any of these ten positions, suggesting that single arginines are not sufficient to improve the existing polymerase ribozyme. This also suggests that it may be harder than previously thought to take the first step in the development of the translation apparatus, via amino acid - nucleic acid conjugates.

## Results

**Figure 1:**
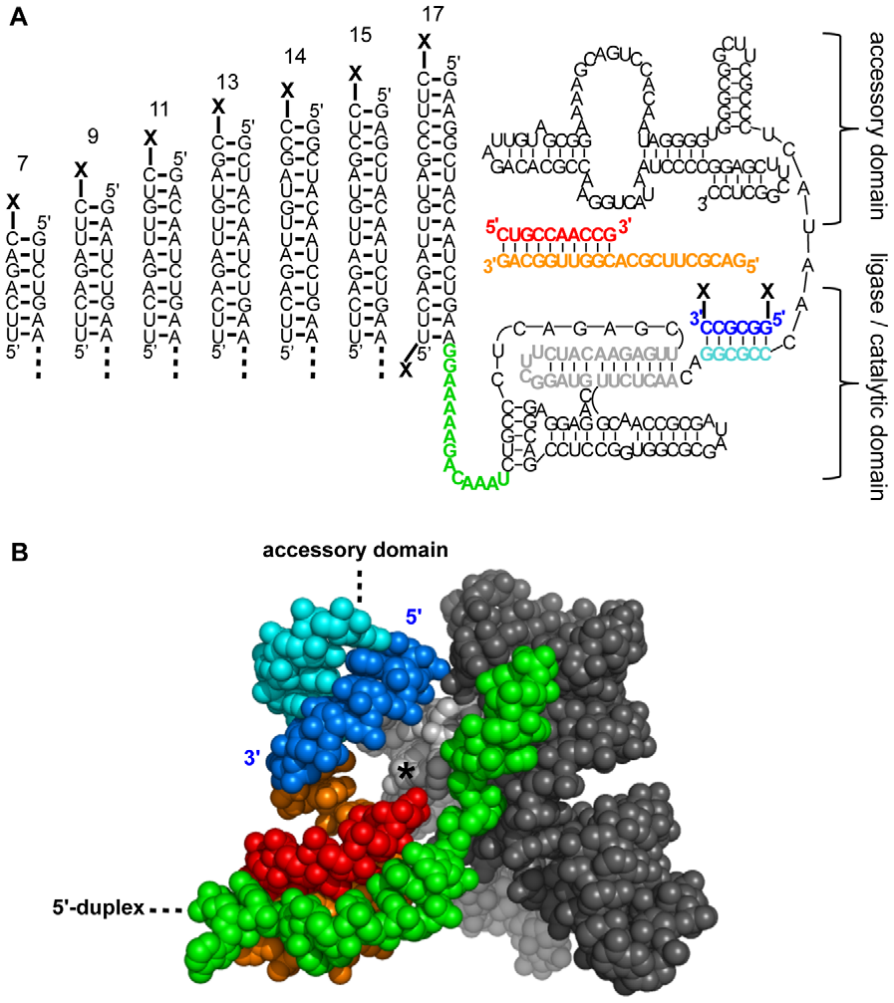
Structure of ribozyme constructs used in this study. The 5′-terminus of the ribozyme (green) is in close contact with the primer (red) and template (orange). The P2 oligo (dark blue) is base paired to a complementary region on the ribozyme (light blue), forming the P2 helix. (**A**) Secondary structure of the polymerase ribozyme [7] with the 5′-duplexes and the P2 duplex that were used to attach arginine or amino cofactors. The length of the 5′-duplex is indicated. “X” denotes the position of the chemical modification. The P2 oligo is truncated, and the internal mismatch was removed [12]. (**B**) 3D structure of the ligase domain in the polymerase ribozyme, based on the crystal structure of the ligase [27]. Atoms that do not appear in the polymerase ribozyme were deleted. The asterisk denotes the position of the catalytic site. The positions where the 5′-duplex and the accessory domain are attached to the ligase domain are indicated, as well as the 5′-terminus and 3′-terminus of the P2 oligo.

We used arginine-RNA and arginine-DNA conjugates to position the arginine cofactor on the ribozyme. Specific sequences for the nucleic acid handle of the conjugate made it possible to base pair the conjugate to different positions on the ribozyme (Fig. 1). This strategy carries several advantages over the use of free amino acids or free peptides. First, a few unpaired bases on the ribozyme are sufficient to base pair to the handle of the conjugate. In comparison, free amino acids or peptides would make it necessary to establish a binding pocket for the cofactors on the ribozyme. Second, the amino acid portion of the conjugate is accessible for interactions with the substrate. In contrast, free amino acids and short peptides require a cofactor-binding pocket that obstructs at least some of the possible interactions with the primer/template.

The arginine-nucleic acid conjugates were synthesized by carbodiimide peptide coupling chemistry. Fmoc-protected arginine was activated as NHS ester and reacted with amino modified DNA or RNA. The nucleic acid sequences of these conjugates were chosen to pair to one of two target sites on the polymerase ribozyme, thereby forming a 5′-terminal duplex, or the P2 duplex. The choice of these target sites was based on their vicinity to the catalytic site (Fig. 1; [27]) and because base pairing to these sequences did not inhibit ribozyme polymerization.

Ten polymerase ribozymes have been developed to date [7], [8], [9], [10]. Our study focuses on the first published polymerase ribozyme [7] because this was the most efficient polymerase ribozyme at the beginning of our study. Our results are relevant for at least the three most efficient variants of these ribozymes because their secondary structure is almost identical [7], [9], [10].

### A 5′-duplex on the polymerase ribozyme to attach arginine conjugates

The first site for attaching the arginine conjugates is a duplex that extends the 5′-terminus of the polymerase ribozyme [19]. Choosing this 5′-duplex for attaching the arginine has the benefit that the length of the duplex can be varied, thereby tethering an arginine to the distal end of the duplex places it at different positions along a ‘helical ruler’ on the ribozyme [28]. Additionally, the proximal end of the duplex accesses another position. Therefore, the 5′-duplex allowed us to place an arginine cofactor at eight positions on the polymerase ribozyme (Fig. 1). In the absence of a complementary RNA or DNA, all single-stranded 5′-terminal ribozyme sequences inhibited polymerization (data not shown), confirming that the conjugates annealed to their intended position at the ribozyme 5′-terminus.

**Figure 2:**
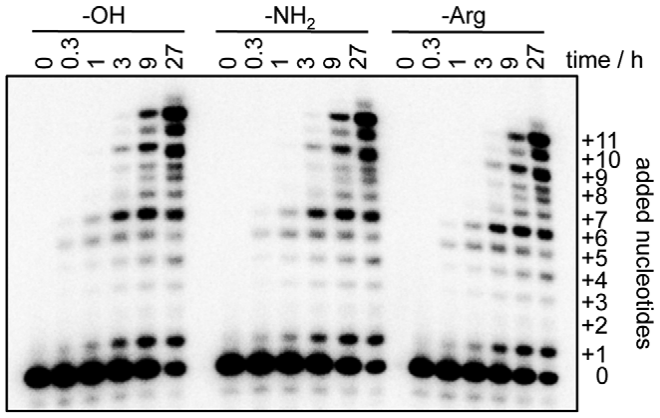
Influence of arginine and amino modifications at the proximal end of the 5′-duplex, on polymerization. Shown is an autoradiogram of PAGE separated polymerization products. For each sample, the polymerization products at six incubation times are shown. The number of nucleotides added to the primer during polymerization is indicated. The eighth nucleotide addition results in two bands due to nucleotide misincorporation. The length of the 5′-duplex was 17 base pairs. No difference in polymerization efficiency was found between unmodified and modified ribozymes, within the errors of three replications of the experiments.

**Figure 3:**
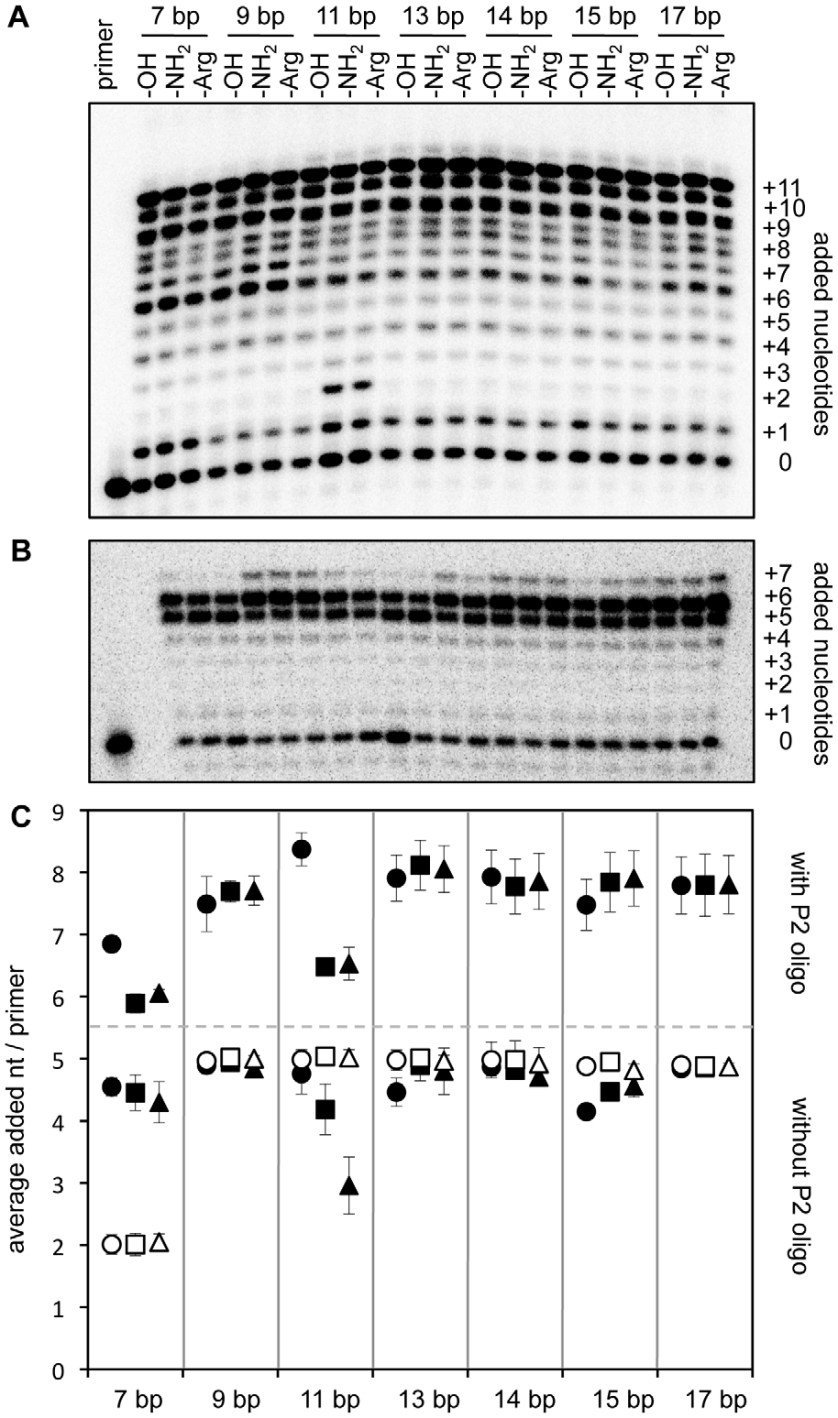
Influence of arginine and amino modifications at the distal end of the 5′-duplex on polymerization. (**A**) Autoradiogram of PAGE separated polymerization products, with RNA/RNA duplexes at the 5′-terminus of the ribozyme, in the presence of our P2 oligo. The length of the 5′-duplexes as well as the chemical modification, are indicated. (**B**) Autoradiogram of PAGE separated polymerization products, with RNA/RNA duplexes at the 5′-terminus of the ribozyme, in the absence of a P2 oligo. The length of the 5′-duplexes as well as the chemical modification, are indicated. (**C**) Quantitation of polymerization efficiencies for RNA/RNA (filled symbols) and DNA/RNA (open symbols) duplexes at the ribozyme 5′-terminus. The polymerization efficiency is described as the average number of nucleotides added per primer. For each length of the 5′-duplex, three variants were tested: Unmodified (circles), amino modified (squares), and arginine modified (triangles) duplexes. Symbols above the grey dashed line show the results of reactions in the presence of the P2 oligo; symbols below the grey dashed line show the results in the absence of a P2 oligo. Errors are standard deviations from three experiments.

To measure the effect of each positioned arginine on ribozyme function we quantified the polymerization efficiency with and without the arginine modification. Additionally, we measured the influence of an amino group, which was used to couple the arginine with the nucleic acid handle. The polymerization efficiency was measured as the average number of nucleotides added to each primer molecule. This readout is sensitive enough to allow the detection of single hydrogen bonds between the ribozyme and the primer/template substrate [14]. However, the arginine modification did not show any effect on ribozyme polymerization when it was placed at the proximal end of the 5′-duplex or at the distal end of the 5′-duplex, with duplex lengths of 9, 14, and 17 base pairs (Fig. 2 and 3).

When the 5′-duplex had a length of 7 base pairs, the DNA derivatives showed lower polymerization efficiency than the RNA derivatives (Fig. 3). This can be explained by the lower thermodynamic stability of DNA/RNA duplexes relative to RNA/RNA duplexes [29]. We assume that the 7-base pair DNA/RNA duplex was partially dissociated so that the single-stranded 5′-sequence of the ribozyme could inhibit polymerization. This interpretation is supported by our observations that the optimal reaction temperature with the 7-base pair DNA/RNA duplex was slightly lower than with longer duplexes, and that the single-stranded 5′-sequence inhibits polymerization (data not shown). All 5′-duplexes longer than 7 base pairs appeared to be stable under the used reaction conditions.

### Inhibitory effects of arginine at the 5′-duplex

When the length of the 5′-duplex was 11 base pairs the arginine and amino modification showed an inhibitory effect on polymerization (Fig. 3C). While this effect occurred both in the absence and the presence of a P2 oligo, an inhibitory effect at the 7 base pair duplex appeared only in the presence of the P2 oligo. The P2 oligo was introduced as a heptanucleotide that complements the P2 duplex on the polymerase ribozyme (Fig. 1A) and improved some aspects of polymerization [7]. However, later studies showed that polymerization also proceeds well in the absence of the P2 oligo [19] and that a truncated P2 oligo is more efficient than the heptanucleotide [12]. Because the P2 oligo binds adjacent to the catalytic site we tested most effects found in this study with and without the optimized, truncated P2 oligo. Because the P2 duplex affected the positioning of the 5′-duplex with 7 base pairs on the ribozyme but not that of longer 5′-duplexes we assume that the ribozyme forms fundamentally different interactions with 5′-terminal duplexes of 7-base pairs and longer duplexes.

### Arginine at the 5′-duplex can rescue inhibitory effects

When the 5′-duplex had a length of 15-base pairs the RNA/RNA duplex slightly decreased polymerization, in the absence of a P2 oligo (Fig. 3C, lower panel). This was concluded from comparing the polymerization efficiency between RNA/RNA duplexes and DNA/RNA duplexes, as well as between the unmodified, amino modified, and arginine modified RNA/RNA duplex. The inhibitory effect of the 2′-hydroxyl group was rescued by the 2′-deoxy modification as well as by the 3′-terminal amino or arginine modification of the RNA. The rescue by 3′-terminal modifications showed that the 3′-terminal 2′-hydroxyl group caused the inhibitory effect and not internal 2′-hydroxyl groups in the RNA/RNA duplex. These effects probably also existed for a duplex length of 13 base pairs and in the presence of the P2 oligo but were too small to have strong statistical significance (Fig. 3C).

### 5′-terminal duplexes can enter the catalytic site

**Figure 4:**
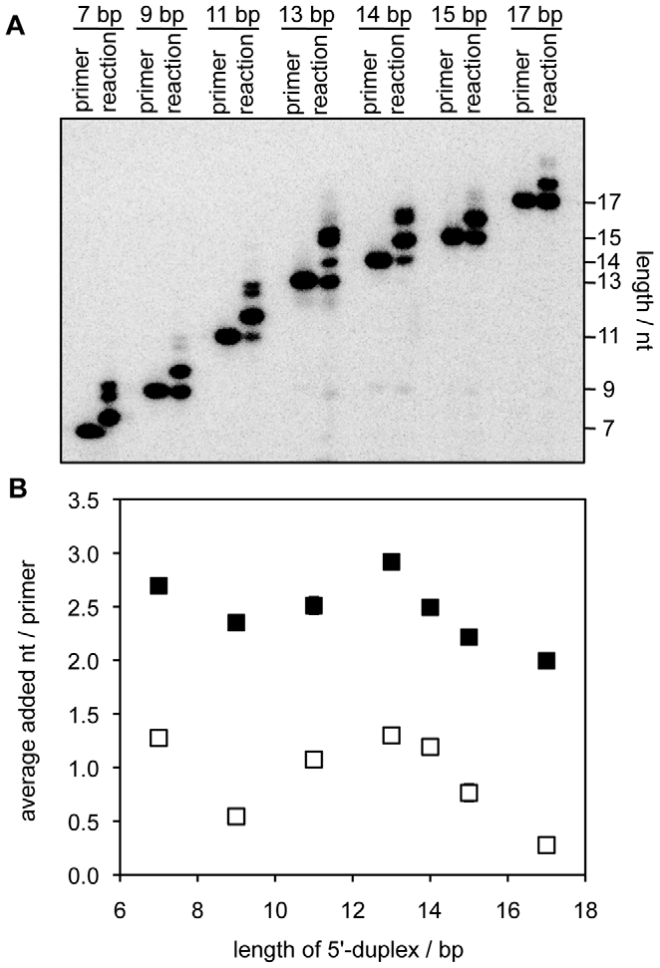
Extension of primers that were base paired to the 5′-terminus of the polymerase ribozyme. The extension efficiency of these primers was measured as a function of the length of the 5′-duplex. (**A**) Autoradiogram of PAGE separated polymerization products, in the absence of the P2 oligo. For each length of the 5′-duplex (indicated) the unreacted primer and the reaction products are shown. (**B**) Quantitation of polymerization efficiencies. The polymerization efficiency was measured as the average number of nucleotides added per primer and plotted as a function of the length of the 5′-duplex. The polymerization efficiencies in the absence (open squares) and in the presence of the P2 oligo (filled squares) are shown. Errors are standard deviations from triplicate experiments and were usually smaller than the symbols.

To explain the inhibitory effect of the 3′-terminal RNA 2′-hydroxyl group at the 5′-duplex we hypothesized that the distal terminus of the RNA/RNA duplex entered the active site and interfered with binding of the primer/template. To test whether the inhibitory effect of the 3′-terminal 2′-hydroxyl group could be due to insertion into the catalytic site we monitored whether the 5′-duplex could be used as a primer/template duplex and extended by the polymerase ribozyme. To obtain a templating sequence the 5′-terminus of the polymerase ribozyme was elongated by four nucleotides. Polymerization assays showed that the radiolabeled RNAs at the 5′-duplex were indeed extended by the polymerase ribozyme, with a strong dependence on the length of the 5′-duplex (Fig. 4). The same length dependence was visible in the absence and the presence of the P2 oligo. The dependence followed a pattern that coincided with the periodicity of an A-form helix (11 base pairs), with the exception of the 7 base pair duplex. These results showed that the distal terminus of the 5′-duplex entered the catalytic site of the ribozyme, confirming the hypothesis that the 3′-terminal 2′-hydroxyl group of 5′-terminal RNA/RNA duplexes could inhibit polymerization by insertion into the catalytic site.

### Arginine at the P2 duplex can mediate an initial burst of polymerization

The second site on the ribozyme that was used for the attachment of conjugates is the P2 duplex, which was formed by the polymerase ribozyme base pairing to the RNA hexanucleotide 5′-GGCGCC-3′ [7], [12] (Fig. 1). The P2 oligo is positioned adjacent to the catalytic site as judged by the crystal structure of the catalytic core of the ribozyme (Fig. 1B; [27]). To test whether the positive charge next to the catalytic site could improve polymerization further we modified both the 5′-terminus and the 3′-terminus of this P2 oligo with arginine (Fig. 1A).

**Figure 5:**
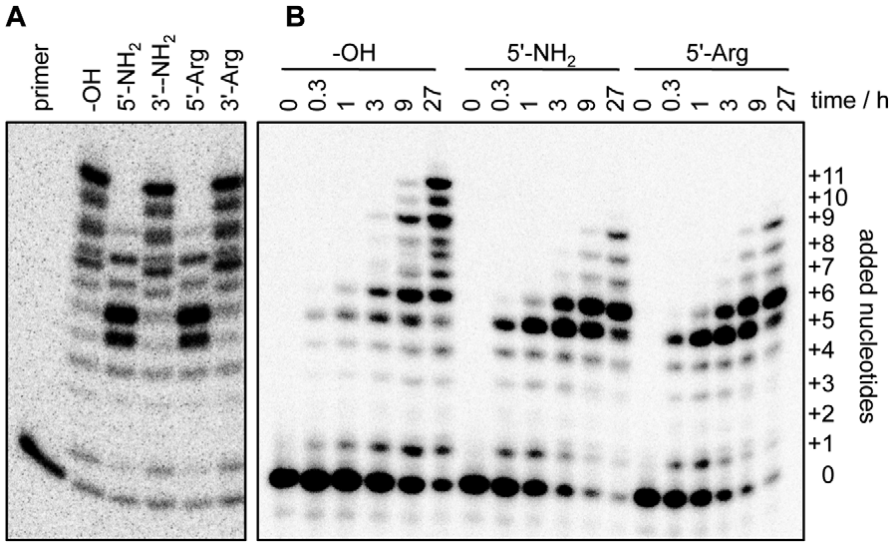
Influence of arginine and amino modifications at the P2 oligo. The position and the modification of the DNA P2 oligo 5′-GGCGCC-3′ is shown for each reaction, as well as the number of nucleotides added to the primer. The images are representative for three experiments. (**A**) Autoradiogram of PAGE separated polymerization products, after 24 hours of polymerization. (**B**) Autoradiogram of PAGE separated polymerization products, after increasing times for polymerization.

An arginine or amino modification at the 5′-terminus of the P2 oligo resulted in an initial burst of polymerization but caused a stalling of polymerization after five or six nucleotides were added (Fig. 5). This mirrors the behavior when the P2 oligo is a heptanucleotide, differing from our hexanucleotide by a 3′-terminal adenosine [12]. This 3′-terminal adenosine interacts with the single-stranded portion of the same template as used in this study (T21) but not with other templates (T50a, T50b). Consistent with that we did not find an influence of modifications at the 5′-terminus of the P2 oligo when other templates were used (T50a or T50c from reference [12]; data not shown). When the arginine or amino modification was placed at the 3′-terminus of the P2 oligo it did not affect polymerization efficiency. This is consistent with a previous study, which found that nucleotide extensions at the 3′-terminus of the P2 oligo are tolerated [12].

### Influence of arginine conjugates at low magnesium concentrations

**Figure 6:**
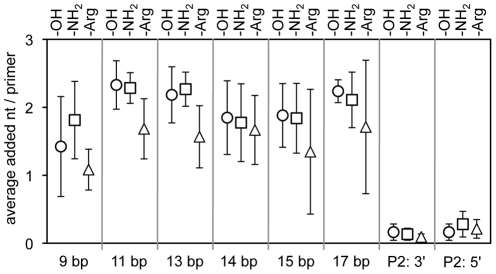
Influence of arginine and amino modifications on polymerization at low magnesium concentration. Quantitation of polymerization efficiencies for DNA conjugates at the ribozyme 5′-terminus (in the absence of a separate P2 oligo) and at the P2 site. The polymerization efficiency is described as the average number of nucleotides added per primer. For each experiment, three DNAs were tested: Unmodified (circles), amino modified (squares), and arginine modified (triangles) DNAs. Errors are standard deviations from three experiments.

All experiments above were conducted at magnesium ion concentrations of 80 mM Mg^2+^ (64 mM free Mg^2+^), which may be too high for arginine to compete with, to bind to phosphodiester oxygens. Therefore, we reduced the concentration of Mg^2+^ to 40 mM (24 mM free Mg^2+^), which is high enough to obtain quantifiable data from polymerization but perhaps low enough to see a positive effect of arginine cofactors [19]. A positive effect by arginine cofactors at this concentration would not mean that the polymerase ribozyme efficiency is improved over its optimal activity (which requires 200 mM Mg^2+^) but that single arginines could have a role in nucleic acid interactions in an RNA world, at these lower Mg^2+^ concentrations. However, even at this low concentration we did not detect increased polymerization efficiencies due to arginine (Fig. 6). On the contrary, the arginine modification was inhibitory when the 5′-duplex had a length of 11 or 13 base pairs. The inhibitory effect at a duplex length of 11 base pairs was consistent with the effect at 80 mM Mg^2+^ whereas the inhibitory effect at a 5′-duplex length of 13 base pairs was not seen at 80 mM Mg^2+^ and may therefore reflect a minor structural change of the ribozyme between 40 mM and 80 mM Mg^2+^.

## Discussion

In an effort to increase the polymerization efficiency of the polymerase ribozyme we tested whether arginine conjugates could improve polymerization. However, we found that arginine did not improve polymerization when placed at ten different positions on the polymerase ribozyme.

Why did the use of arginine-nucleic acid conjugates not improve the efficiency of the polymerase ribozyme? One possibility is that a single arginine is not sufficient to show a strong effect on primer/template binding. However, our assay is sensitive enough to detect even single hydrogen bonds that affect substrate binding [14]. Second, although we tested ten different positions for arginine on the ribozyme the best location may not have been among them. Third, the current polymerase ribozymes may not benefit from the conjugates because the ribozymes were optimized in the absence of these conjugates. A partial randomization and re-selection in the presence of these conjugates may find polymerase ribozymes that efficiently use the conjugates. Lastly, it is possible that the magnesium concentration that is necessary for activity of the polymerase ribozyme shielded the phosphodiester groups sufficiently that the effect of an arginine was too low to detect. Although we decreased the concentration of free magnesium ions to 24 mM (Fig. 6) we did not find a beneficial effect of arginine on polymerization. This suggests that at the magnesium concentration necessary for activity of the ribozyme single arginines cannot improve polymerization of the existing polymerase ribozymes.

The potential benefit of the amino acid histidine for acid-base catalysis in ribozymes and deoxyribozymes was explored previously. Histidine promised to be useful for a catalytic function because it has a pK_A_ close to the neutral pH, whereas nucleic acids do not [30]. Indeed, an in vitro selection found deoxyribozymes that use free histidine as cofactor, probably with a catalytic role [31]. However, the rate enhancements of histidine-using deoxyribozymes are not higher than those that use divalent metal ion cofactors or no cofactors at all [32], [33], and it was found that ribozymes can perturb the pK_A_s of nucleobases close to the neutral pH [34], [35], [36]. Additionally, it appears easier for nucleic acids to use divalent cations rather than histidine as cofactor [37]. Therefore, histidine (and perhaps any other amino acid) does not seem to be important for general acid-base catalysis in ribozymes or deoxyribozymes.

Peptides and proteins fulfill several non-catalytic roles in natural ribozymes. The bacterial RNase P ribozyme requires the C5 protein for recognition of the pre-tRNA substrate [38] and ribosomal proteins fulfill a very diverse set of functions [39]. Although natural hammerhead ribozymes do not require a protein cofactor, a trans-acting variant of the hammerhead ribozyme benefits from the nonspecific binding of the HIV p7 nucleocapsid protein, for the annealing of substrates and the dissociation of products [40]. Therefore, trans-acting ribozymes can benefit from peptides or proteins for the function of substrate interactions. Although our study did not identify how single arginines can assist ribozyme polymerization we assume that a different setup with short peptides can help the polymerase ribozyme to bind the primer/template substrate.

Ribozymes have been selected previously to require the presence of a peptide or protein to be active [41], [42], [43]. Here, the peptides/proteins appear to stabilize the catalytically active structure of the ribozyme. However, in none of these cases was the activity of the RNP complex higher than that of the parent ribozyme. Therefore, these RNPs show how the activity of a ribozyme can be regulated by a peptide but not how the activity can be increased. In contrast, our study aimed solely to obtain ribozymes with higher efficiency.

In an RNA world, amino acid - nucleid acid conjugates could have been crucial intermediates for establishing a translation system [20], [21], [24]. In the first step amino acid-nucleic acid conjugates would have been synthesized. Although we did not find a functional benefit of single amino acid conjugates for the polymerase ribozyme they could have carried different immediate evolutionary advantages [20]. With respect to the evolution of the translational apparatus these conjugates would have served as the ancestors of aminoacyl-tRNAs [44]. The next step in the evolution would have been the attachment of multiple amino acids to a single conjugate. This formation of peptide bonds can be catalyzed by ribozymes [45] and could have carried immediate benefits, for example by tighter interactions of diarginine with RNA than of arginine. If the source of this second amino acid would have been another conjugate then the ribozyme that catalyzed this peptidyl transfer would have been a primitive ribosome: the conjugates (precursors of tRNAs) would be aligned by base pairing to an mRNA (either a sequence in the ribozyme or a separate RNA). The nucleic acid portion of the conjugate would then have served as precursor to the tRNA anticodon and facilitated the first encoded peptide synthesis. Further evolutionary steps would have improved the efficiency and accuracy of this machinery to the present-day translation apparatus. One strength of this model is that each of these evolutionary steps has been shown to be accessible to ribozymes, and that each evolutionary step carried an evolutionary advantage for the RNA world organism [20], [46].

## Materials and Methods

### Ribozymes and substrates

Ribozymes were synthesized by in vitro transcription from PCR products, using bacteriophage T7 RNA polymerase as described [19]. Transcribed ribozymes were purified by 7 M urea 5% polyacrylamide gel electrophoresis (PAGE). RNAs were purchased from Dharmacon, and DNAs were purchased from IDT. All RNAs and DNAs were PAGE purified. Primers were radiolabeled using T4 polynucleotide kinase (NEB) and [γ-^32^P] ATP (Perkin-Elmer). All chemicals were Molecular Biology grade or higher.

### Synthesis of conjugates

Arginine conjugates were synthesized from amino-modified RNAs or DNAs via NHS-activated arginine. The amino modifications contained a tether to the nucleic acid by six methylene groups (C6-linker). In dry DMF, 143 mM a-amino-Fmoc-arginine were reacted with 143 mM N-hydroxy succinimide (NHS) and 143 mM N,N′-dicyclohexyl carbodiimide (DCC) for 1 hour at 50°C. After cooling to room temperature the reaction mixture was mixed with the 2.5-fold volume of an aqueous solution with 100 mM amino-modified nucleic acid and 200 mM MES/NaOH pH 6.5. After incubation for 2 hours at room temperature the reaction mixture was dried in vacuum, then deprotected in an excess of 100 mM NaOH for 1 hour at 40°C. RNA coupling products were deprotected in an excess of 50 mM NaOH and 50 mM Na_2_CO_3_ for 2 hours at 10°C. Deprotected products were neutralized, ethanol precipitated, and purified by 7 M urea 20% PAGE. The overall yield was 5–10% for RNA-arginine conjugates and 10–20% for DNA-arginine conjugates, as calculated based on the nucleic acid. The identity of the DNA conjugates was confirmed by MALDI Mass Spectroscopy, using hydroxypicolinic acid and ammonium citrate as matrix and a DNA 12mer and 21mer as internal standard for calibration. Expected mass for our test amino modified DNA: 5330.6; found: 5330.7. Expected mass for the corresponding arginine modified DNA with Fmoc protection: 5709.0; found: 5708.7. Expected mass for deprotected arginine modified DNA: 5486.8. Found: 5486.9. Additionally, the identity of both DNA and RNA conjugates was confirmed by the migration pattern of 5′-radiolabeled samples in denaturing PAGE.

### Ribozyme Reactions

Ribozyme reactions were performed as described [19]. All RNAs were dissolved in water at the appropriate concentration (final reaction concentration: 2 µM Ribozyme, 1 µM template, less than 50 nM 5′-radiolabeled primer, 2.5 µM P2 oligo, 2.5 µM 5′ terminus oligo), heat denatured (2 min/80°C) and cooled to the reaction temperature (17°C) at 0.1°C/sec. Reactions were started by adding 2.5× reaction buffer containing magnesium chloride, buffer (Tris/HCl, pH 8.5), and NTP (an equimolar mix of the four nucleoside triphosphates) so that the final concentrations were 50 mM Tris/HCl and 4 mM of each NTP. Magnesium chloride was 80 mM with the exception of primer extensions at the 5′-duplex (200 mM MgCl_2_), or reactions annotated to contain 40 mM MgCl_2_. Reaction times were 24 hours for reactions with P2 oligo and 3 hours for reactions without P2 oligo if not indicated otherwise. Reaction times for reactions with 40 mM MgCl_2_ were 22 hours. The reason for the different incubation time is that in the absence of the P2 oligo polymerization is fast during the first hours and then stalls, whereas in the presence of the P2 oligo polymerization is slower during the first hours but extends further [12]. The reactions were stopped by the addition of a 1.5 fold volume of stop buffer (80% (v/v) formamide, 200 mM Na_2_EDTA at pH 8.4) and a template-complementary RNA added in 20-fold excess over the template. The mixtures were heat denatured (2 min/80°C) and cooled to room temperature at 0.1°C/sec before loading and separating on 7 M urea 0.5× TBE 20% PAGE.

### Data analysis

Autoradiographs of the PAGE separations were recorded by a PMI phosphorimager (Bio-Rad) and quantitated using the software Quantity One. Shifts higher than 11 nt above the primer were counted as full-length extension. The values for “average nucleotides per primer” were obtained by multiplying the fraction of intensity for each band (minus background signal) with the number of added nucleotides corresponding to that band. For quantifying the effect of 2′-deoxy substitutions, the method was described previously [14]. All experiments were repeated at least in triplicate.
